# Preoperative Diagnosis of Symptomatic Adenomyosis: Limitations and Clinical Insights

**DOI:** 10.7759/cureus.100218

**Published:** 2025-12-27

**Authors:** Manuela Pereira, Swati Kumari, Lucia Di Francesco, Salma Moustafa, Liaisan Uzianbeava, Pengfei Wang

**Affiliations:** 1 Obstetrics and Gynecology, BronxCare Health System, Bronx, USA

**Keywords:** abnormal uterine bleeding, adenomyosis, dysmenorrhea, dyspareunia, mri, tvus, uterine fibroids, uterine tenderness

## Abstract

Objectives: To assess the diagnostic accuracy of adenomyosis on transvaginal ultrasound (TVUS) in a community hospital.

Design: Retrospective cohort study

Participants: A total of 124 patients who underwent hysterectomy between September 2022 and August 2024.

Setting: A tertiary referral academic center

Methods: All patients were referred to the Fibroid Clinic (BronxCare Health System) with symptomatic uterine fibroids or adenomyosis and underwent total hysterectomy. Postoperative pathological results were used as the reference standard. Collective data on TVUS, history, physical examination, and preoperative diagnosis were all studied. The primary goal was to evaluate the accuracy of TVUS in diagnosing adenomyosis by either radiologists or minimally invasive gynecologic surgeon (MIGS). The secondary objective was to identify the clinical factors associated with preoperative diagnosis of adenomyosis.

Result: According to histologic reports from the surgical specimen, 61 (49.2%) patients had uterine fibroids only, 50 (40.3%) had concomitant fibroids and adenomyosis, and 13 (10.5 %) had adenomyosis only. There were no significant differences in age, race, or BMI among patients in the three groups. The accuracy of radiologist-interpreted TVUS for adenomyosis was found to be low in patients with pure adenomyosis (0 out of 13) and in those with both fibroids and adenomyosis (0 out of 50). In contrast, MIGS interpretation demonstrated 83.3% sensitivity for pure adenomyosis, which decreased to 30.6% in the presence of concomitant fibroids. The presence of uterine tenderness (UT) on bimanual examination is an independent predictor of adenomyosis, even with the presence of uterine fibroids or a uterine size larger than 14 weeks. When the uterine size was less than 14 weeks, the clinical presence of dysmenorrhea, dyspareunia, or UT was significantly associated with adenomyosis. The clinical assessment and TVUS interpretation by MIGS resulted in a 62.0% sensitivity and 84.0% specificity for adenomyosis in our cohort.

Conclusion: The diagnosis of adenomyosis based on TVUS or a radiologist’s interpretation of TVUS can be underestimated. A comprehensive assessment, including clinical history, physical examination, and ultrasound interpretation by gynecologists, is essential for accurate diagnosis, patient counseling, and appropriate management.

## Introduction

Adenomyosis is a benign uterine disease, defined by Dr. Bird in 1972 as a benign invasion of endometrium into the myometrium, producing a diffusely enlarged uterus. Microscopically, this disorder exhibits ectopic non-neoplastic endometrial glands and stroma surrounded by hypertrophic and hyperplastic myometrium [1]. The major symptoms of adenomyosis are abnormal uterine bleeding (AUB), dysmenorrhea, dyspareunia, and adverse reproductive outcomes, including subfertility and pregnancy complications [2]. The true prevalence of adenomyosis is unknown and has been estimated to range from 8.8% to 61.5% based on hysterectomy data in the past 50 years [3]. Over the past one to two decades, non-invasive diagnosis of adenomyosis using transvaginal ultrasound (TVUS) and MRI has been well studied and widely integrated into clinical practice. Multiple meta-analyses and systematic reviews demonstrated that the sensitivity and specificity of TVUS range from 65% to 85% and 43% to 97.5%, respectively [4-8]. MRI has been reported to have similar rates, with sensitivities ranging from 71% to 78% and specificities from 88% to 92.5% [5,7,8]. Therefore, the 2D or 3D pelvic ultrasound is recommended as the first line of modality for adenomyosis, due to its low cost, wide availability, and high rate of sensitivity and specificity [4,5,7,8]. Ultrasound features for adenomyosis by using the morphological uterine sonographic assessment (MUSA) were first defined in 2015 and subsequently revised in 2022 [9,10]. In the 2022 consensus, experts agreed that MUSA features should be divided into direct and indirect ultrasound signs of adenomyosis. Direct features indicate the presence of ectopic endometrial tissue in the myometrium, including myometrial cysts, hyperechogenic islands, and echogenic subendometrial lines and buds. Indirect features are those that are secondary to the presence of endometrial tissue in the myometrium, including a globular uterus, asymmetrical myometrial thickening, fan-shaped shadowing, translesional vascularity, irregular junctional zone (JZ), and interrupted JZ [10]. Expert use of TVUS has greatly advanced the understanding of adenomyosis. One striking finding is that its prevalence in women under age 30 presenting with dysmenorrhea or heavy menstrual bleeding may reach 27.4%, according to TVUS [11]. This is different from the traditional belief that adenomyosis most commonly happens to women in the late premenopausal stage, an assumption rooted in histological diagnoses from hysterectomies, which are most commonly performed in women in their 40s [3].

TVUS is an excellent modality for adenomyosis; however, we need to interpret the data from publications prudently. Outside of controlled research environments, radiology reports may have substantially lower sensitivity for adenomyosis than suggested in the literature or expected by clinicians. A recent study reported that the sensitivity of pelvic ultrasound for adenomyosis was only 10.9% and the sensitivity of MRI was only 29.7% in the real practice setting [12]. Another study also reported that the inter-observer and intra-observer agreement on different MUSA features was only moderate, which indicated the lack of consensus in detecting adenomyosis even among radiologists [13]. Another concern is that the ultrasound is performer-dependent. A recent study demonstrated that gynecologic expert sonologists were 7.8 times more likely to detect adenomyosis than general radiologists (odds ratio (OR) = 7.84; 95% confidence interval (CI), 4.58−13.44) [14]. At last, multiple studies showed that the concomitant presence of uterine fibroids can significantly decrease the accuracy of ultrasound for adenomyosis [14,15]. Taken together, these findings suggest that adenomyosis is more likely to be underdiagnosed and undertreated in routine clinical practice. Further research is urgently needed to improve preoperative diagnostic methods for adenomyosis.

In this study, we report our experience in managing patients with adenomyosis in a community hospital. Our findings highlight the limited accuracy of radiologist-interpreted TVUS in real world practice in diagnosing adenomyosis and emphasize the importance of clinical judgment by gynecologists in guiding diagnosis and treatment of coexistent uterine disorders.

## Materials and methods

Design of the study and patient population

This study is a single-center, retrospective cohort study. All patients were referred to the Fibroid Clinic (BronxCare Health System) with presumed symptomatic uterine fibroids. Hysterectomies were performed at a single hospital between September 2022 and August 2024, based on a preoperative diagnosis of uterine fibroids or adenomyosis. The study was approved by the Institutional Ethics Committee. The primary objective was to evaluate the diagnostic accuracy of TVUS for adenomyosis. The secondary objective was to identify clinical parameters associated with a preoperative diagnosis of adenomyosis.

A comprehensive chart review was conducted. Patient demographics, including age, race, and BMI, were collected. Symptoms assessed included AUB, dysmenorrhea, dyspareunia, bulk symptoms, infertility, and chronic pelvic pain (CPP). Physical examination findings, specifically uterine size and uterine tenderness (UT), were also reviewed. Histopathologic reports from surgical specimens served as the reference standard for diagnosing fibroids and adenomyosis.

TVUSs were conducted at a single institution by ultrasound technicians. Four radiologists interpreted the ultrasound images and signed the reports for the clinicians. A single minimally invasive gynecologic surgeon (MIGS) reviewed the TVUS independently. The interpretations of the TVUS and the preoperative diagnoses were documented in the patient charts. All hysterectomy surgeries were performed by the same MIGS. The experience of the four radiologists varies from 5 to 25 years. The experience of MIGS is about 10 years.

Statistical analysis

The normal distribution of the data was verified using the Kolmogorov-Smirnov test. Continuous quantitative variables were expressed as median (interquartile range (IQR)) or as mean (± SD) when appropriate. Categorical variables were expressed as counts (no.) and %. The main outcome was the presence of histologically confirmed adenomyosis. The comparison of continuous variables between groups was assessed using the Mann-Whitney U or Kruskal-Wallis tests, as appropriate. The comparison of the categorical variables was carried out using the Chi-square test or Fisher's exact test. Binary regression analysis was used to investigate predictors for the presence of adenomyosis. The diagnostic performance of clinical variables and imaging was evaluated. Linear regression analysis was used to assess the relationship between variables when appropriate. A p-value < 0.05 was considered statistically significant. The statistical analysis was carried out using Statistical Package for the Social Sciences (SPSS) software 26.0.

## Results

Patient population and characteristics

**Figure 1:**
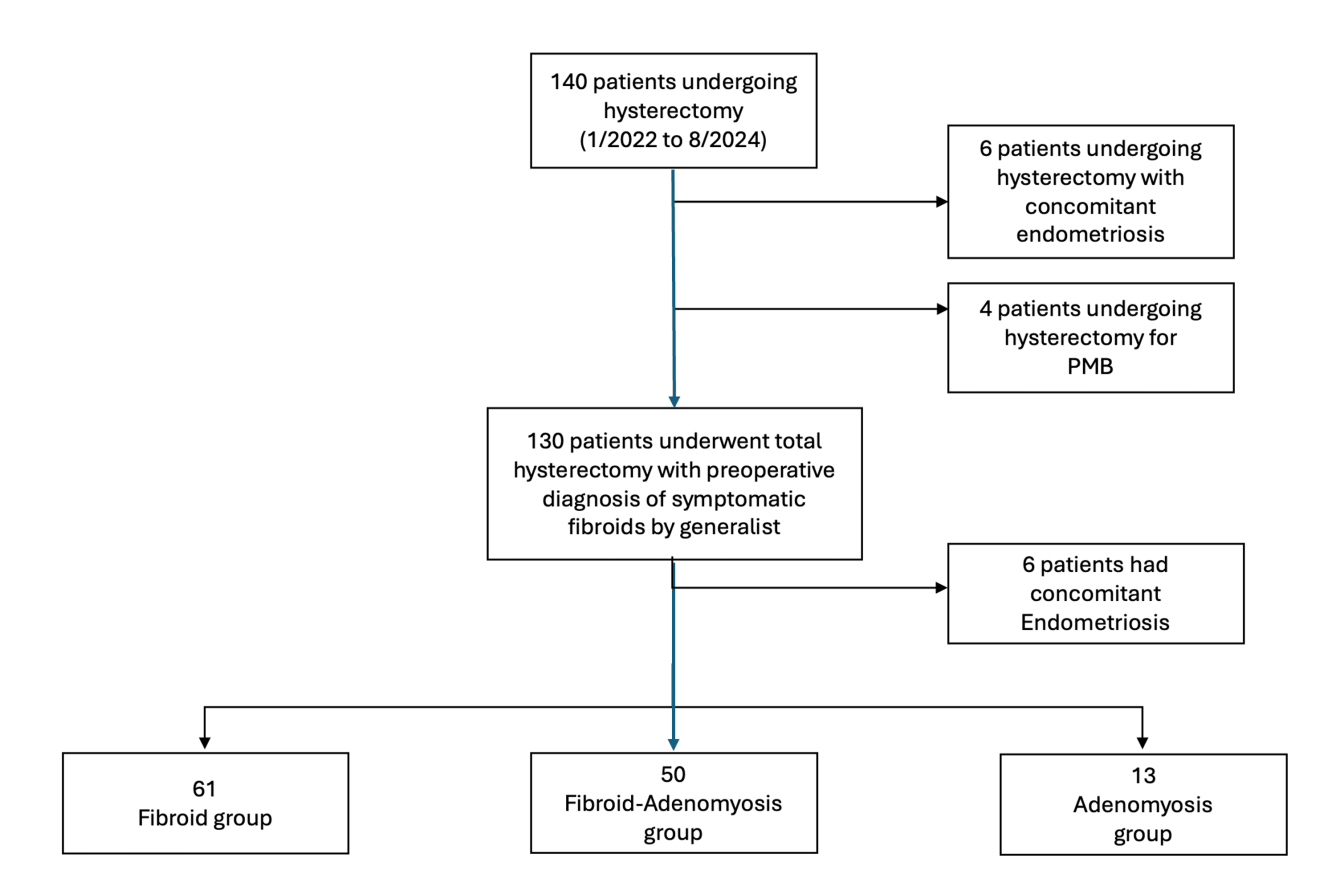
The flow of data collection and group allocation based on the pathology report

A total of 140 patients underwent hysterectomy between September 2022 and August 2024, and 124 cases were included in this study. Ten patients were excluded due to indications of endometriosis or postmenopausal bleeding. An additional six were excluded because of intraoperative findings of endometriosis. The remaining 124 patients were divided into 3 groups based on the final pathology report: “fibroid group” (n=61), “fibroid/adenomyosis group” (n=50), and “adenomyosis group” (n=13) (Figure 1).

There were no significant differences in age, BMI, or race among the three groups. However, statistically significant differences were observed in the prevalence of dysmenorrhea (52.5% vs. 64.0% vs. 100%, P < 0.05) and dyspareunia (27.9% vs. 36.0% vs. 78.9%, P < 0.01) in fibroid, fibroid/adenomyosis and adenomyosis groups, respectively. No significant differences were found for AUB, CPP, or bulk symptoms across the groups. Physical examination findings revealed significant differences in the uterine size between the groups (16.0 vs. 14.0 vs. 12.0 weeks’ size; P < 0.01) and UT (11.5% vs. 36.0% vs. 84.6%; P < 0.01), respectively. Importantly, preoperative uterine size by clinical examination is correlated well with uterine weight, as reported by pathology (R 0.897, P<0.001) (Table 1).

**Table 1:** Clinical characteristics of the study groups AUB: Abnormal uterine bleeding; CPP: Chronic pelvic pain; UT: Uterine tenderness; TVUS: Transvaginal ultrasound; TLH: Total laparoscopic hysterectomy, TRH: Total robotic hysterectomy; TAH: Total abdominal hysterectomy

Parameter	Fibroid (n=61)	Fibroid/Adenomyosis (n=50)	Adenomyosis (n=13)	P-Value
Age (years)	46.0 (44.0-48.0)	46.5 (44.3-49.0)	45.0 (44.0-47.0)	0.138
Races-No. (%)				0.96
Hispanic	41.0 (65.1%)	31.0 (61.0%)	9.0 (69.2%)	
African American	20.0 (31.7%)	18.0 (36.0%)	4.0 (30.8%)	
White	1 (1.6%)	0	0	
Others	1 (1.6%)	1.0 ( 2.0%)	0	
BMI (kg/m^2^)	30.3 (27.1-33.2)	29.9 (27.7-34.2)	28.9 (25.9-32.0)	0.296
Symptoms-No. (%)				
AUB	56.0 (91.8%)	46.0 (92.0%)	12.0 (92.3%)	0.998
Bulk	7.0 (11.5%)	4.0 (8.0%)	0	0.402
Dysmenorrhea	32.0 (52.5%)	32.0 (64.0%)	13.0 (100%)	0.005
Dyspareunia	17.0 (27.9%)	18.0 (36.0%)	10.0 (76.9%)	0.004
CPP	11.0 (18.0%)	12.0 (24.0%)	4.0 (38.0%)	0.531
Infertility	0	1.0 (2.0%)	0	0.474
Physical examination				
Uterine size (weeks)	16.0 (15.0-18.0)	14.0 (13.0-18.0)	12.0 (9.0-12.0)	<0.001
UT-No. (%)	7.0 (11.5%)	18.0 (36.0%) a	11.0 (84.6%)	<0.001
Imaging-No. (%)				
TVUS	60.0 (98.4%)	49.0 (98.0%)	12.0 (92.3%)	0.492
MRI	20.0 (32.8%)	17.0 (34.0%)	2.0 (15.4%)	0.415
Surgical mode-No. (%)				0.523
TLH	26.0 (42.6%)	19 (38.0%)	5.0 (38.5%)	
TRH	27.0 (44.3%)	27 (54.0%)	8.0 (61.5%)	
TAH	8.0 (13.1%)	4 (8.0%)	0	

Of the 124 patients, 121 underwent TVUS and 39 underwent MRI before surgery. There were no significant differences in imaging modality selection among the three groups. Regarding surgical approach, 50 out of 124 patients underwent laparoscopic hysterectomy, 62 underwent robotic-assisted hysterectomy, and 12 underwent abdominal hysterectomy. There were no significant differences in surgical approach among the three groups (Table 1). 

Accuracy of TVUS for adenomyosis

In the fibroid group, 60 out of 61 patients underwent TVUS. Of these, 59 were correctly reported as fibroids and 1 was reported as normal by radiologists. No cases of adenomyosis were reported by radiologists, but there were 10 cases incorrectly diagnosed as adenomyosis by MIGS.

In the fibroid/adenomyosis group, 49 out of 50 patients underwent TVUS. Radiologists reported 48 cases as fibroids and one as normal, with no reports of adenomyosis. In contrast, MIGS identified concomitant adenomyosis in 15 out of 49 cases.

In the adenomyosis group, 12 out of 13 patients had TVUS. Radiologists reported 10 cases as fibroids and two as normal. MIGS identified adenomyosis in 10 out of 12 cases. Based on these findings, the sensitivity of radiologist-interpreted TVUS for adenomyosis was 0%, while the specificity was 98.3%. In contrast, the sensitivity of MIGS-interpreted TVUS for pure adenomyosis was 83.3%, decreasing to 30.6% when adenomyosis coexisted with fibroids. The overall specificity for MIGS interpretation was 83.3% (Table 2).

**Table 2:** TVUS interpretation by radiologist vs MIGS TVUS: Transvaginal ultrasound; MIGS: Minimally invasive gynecologic surgeon

Parameter	Fibroid (61)	Fibroid/ Adenomyosis (50)	Adenomyosis (13)	Sensitivity (Adenomyosis)	Sensitivity (Adenomyosis/Fibroid)	Specificity
TVUS (Radiology)	60	49	12	NA	NA	NA
Fibroid	59	48	9	NA	NA	NA
Normal	1	1	3	NA	NA	NA
Adenomyosis	0	0	0	0	0	98.3%
TVUS (MIGS)	60	49	12	NA	NA	NA
Fibroid	58	40	0	NA	NA	NA
Normal	0	0	2	NA	NA	NA
Adenomyosis	10	15	10	83.3%	30.6%	83.3%

Predictors of adenomyosis with or without fibroids

Clinical assessment included both patient history and physical examination. Compared to the patient with fibroids only, the patients with adenomyosis more frequently presented with dysmenorrhea (OR 2.27, 95% CI: 1.08-4.76, P = 0.029), uterine size less than 14 weeks (OR 5.62, 95% CI: 2.54-12.41, P < 0.001), and UT (OR 6.58, 95% CI: 2.60-12.68, P < 0.001) (Table 3).

**Table 3:** Clinical presentations related to adenomyosis AUB: Abnormal uterine bleeding; CPP: Chronic pelvic pain; UT: Uterine tenderness; OR: Odds ratio; CI: Confidence interval

Parameter	OR	95% CI	P-value
Symptoms			
AUB	1.04	0.28-3.77	0.958
Bulk	0.52	0.15-1.89	0.316
Dysmenorrhea	2.27	1.08-4.76	0.029
Dyspareunia	2.07	0.98-4.38	0.055
CPP	1.55	0.65-3.67	0.321
Infertility	1.02	0.99-1.05	0.323
Physical Examination			
Uterine size (≤14 vs >14 weeks)	5.62	2.54-12.41	<0.0001
UT	6.58	2.60-12.68	<0.0001

In the multivariate analysis, UT (OR 7.14, 95% CI: 2.04-25.00, P = 0.002) was the only clinical parameter significantly associated with a diagnosis of adenomyosis (Table 4).

**Table 4:** Independent predictors for the preoperative diagnosis of adenomyosis AUB: Abnormal uterine bleeding; CPP: Chronic pelvic pain; UT: Uterine tenderness; OR: Odds ratio; CI: Confidence interval

Parameter	OR	95% CI	P Value
Age	1.05	0.947-1.173	0.337
Race			
Hispanic	0.59	0.033-10.423	0.717
African American	0.71	0.035-14.315	0.824
BMI (kg/m^2^)	1.01	0.931-1.093	0.837
Symptoms			
AUB	3.51	0.27-45.27	0.336
Bulk	2.96	0.24-35.84	0.395
Dysmenorrhea	0.75	0.25-2.18	0.591
Dyspareunia	1.42	0.46-4.40	0.547
CPP	1.62	0.53-4.97	0.398
Physical examination			
Uterine size	0.95	0.86-1.04	0.264
UT	0.14	0.04-0.49	0.002

Diagnostic performance of clinical findings and imaging in the identification of adenomyosis

**Figure 2:**
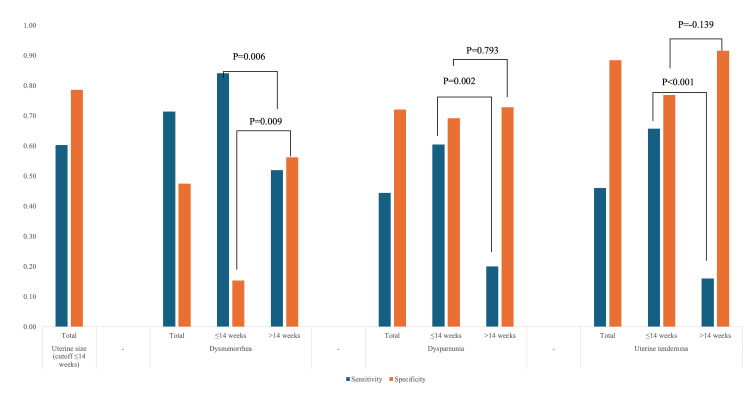
Uterine size less than 14 weeks increases the sensitivity of the clinical presentation of adenomyosis

When differentiating adenomyosis from fibroids clinically, uterine size can serve as a significant confounder, thus the interaction of uterine size and symptoms for the diagnosis of adenomyosis was tested. In this context, the diagnostic performance of dysmenorrhea, dyspareunia, and UT were reassessed according to a cutoff of uterine size of 14 weeks. Accordingly, while sensitivity, positive predictive value (PPV), and positive likelihood ratio (LR+)) improved in patients with uterine sizes ≤14 weeks, the specificity, negative predictive value (NPV), and negative likelihood ratio (LR−) improved in uterine sizes >14 weeks (Table 5). When the uterine size was less than 14 weeks, the accuracy of clinical diagnosis in our cohort was as such: sensitivity 62%, specificity 84%, PPV 80%, NPV 68%, LR+ 3.78, and LR− 0.46 (Table 5, Figure 2).

**Table 5:** Uterine size is a critical factor in assessing the clinical presentation of adenomyosis. UT: Uterine tenderness; MIGS: Minimally invasive gynecologic surgeon; PPV: Positive predictive value; NPV: Negative predictive value; LR+: Positive likelihood ratio; LR-: Negative likelihood ratio

Parameter	Sensitivity	Specificity	PPV	NPV	LR+	LR-
Uterine size ≤14 weeks (n=124)	0.60	0.79	0.75	0.66	2.83	0.50
Dysmenorrhea (n=124)	0.71	0.48	0.62	0.62	1.36	0.60
Dysmenorrhea in ≤14 weeks (n=51)	0.84	0.15	0.74	0.25	1.00	1.03
Dysmenorrhea in >14 weeks (n=73)	0.52	0.56	0.38	0.69	1.19	0.85
Dyspareunia (n=124)	0.44	0.72	0.62	0.56	1.59	0.77
Dypareunia in ≤14 weeks (n=51)	0.61	0.69	0.85	0.38	1.97	0.57
Dypareunia in >14 weeks (n=73)	0.20	0.73	0.28	0.64	0.74	1.10
UT (n=124)	0.46	0.89	0.81	0.61	4.01	0.61
UT in ≤14 weeks (n=51)	0.66	0.77	0.89	0.43	2.85	0.44
UT in >14 weeks (n=73)	0.16	0.92	0.50	0.68	1.92	0.92
Clinical suspicion-MIGS (n=124)	0.62	0.84	0.80	0.68	3.78	0.46

## Discussion

Adenomyosis was first described by Dr. Karl Van Rokitansky in 1860, but it is considered an under-recognized condition and remains under-diagnosed in routine clinical practice [2]. Although advances in molecular research have enhanced our understanding of adenomyosis pathogenesis, and improvements in imaging and hormonal therapies have supported diagnosis and treatment, many clinical and diagnostic challenges remain [16]. This study, conducted at a community hospital, reflects the real-world management of adenomyosis in general gynecology practice.

Although uterine fibroids and adenomyosis often coexist, they are distinct disease entities with different pathophysiology, clinical presentation, treatment options, and prognostic implications [3,17,18]. Similar to endometriosis, adenomyosis is considered more of an inflammatory disease, predominantly presenting as pain, heavy bleeding, and infertility. Adenomyosis can also present as urinary tract symptoms (frequency, urgency, dysuria), gastrointestinal (GI) symptoms (bloating, nausea, constipation, diarrhea, dyschezia, etc.), and CPP [19]. As a result, hormonal suppression may be more effective for adenomyosis than for fibroids. In contrast, uterine artery embolization (UAE) may be less effective, and myomectomy is generally not appropriate for treating symptomatic adenomyosis [19,20]. Given that patients with adenomyosis may develop central or peripheral sensitization, minimally invasive definitive treatment may offer the best relief for pain and improve quality of life. For patients whose primary concern is infertility, accurate clinical diagnosis of adenomyosis is especially important. Many of these patients are initially diagnosed with unexplained infertility and undergo repeated ovulation induction. Identifying adenomyosis as an underlying cause allows providers to offer appropriate treatment, most often in vitro fertilization (IVF) [2,21]. Even when assisted reproductive technologies (ART) are not pursued, a confirmed diagnosis helps patients understand their reduced likelihood of spontaneous conception and may guide them toward hormonal or surgical interventions to improve quality of life. Thus, accurate clinical diagnosis of adenomyosis is essential for both counseling and guiding individualized management plans.

Multiple studies have demonstrated the utility of TVUS and MRI in diagnosing adenomyosis. However, a limitation is the lack of consensus among general radiologists regarding the interpretation of adenomyosis features on imaging [22]. This finding should be interpreted cautiously, given the small number of pure adenomyosis cases and the retrospective, single-center design. While this finding may appear striking, it aligns with existing literature. For example, a separate study from a large academic center found that the radiologic reporting rate for adenomyosis on TVUS was as low as 10.9% when no clinical information was provided. This rate rose significantly to 53.0% when radiologists were explicitly asked to evaluate for adenomyosis [12]. These observations highlight the diagnostic challenge of adenomyosis on imaging and the potential impact of clinical context on image interpretation. Supporting these observations, our study demonstrated that MIGS correctly identified adenomyosis in 10 out of 12 cases with pure adenomyosis, and 15 out of 50 cases with concomitant fibroids and adenomyosis on TVUS. In routine practice, many gynecologic providers are not formally trained in pelvic ultrasound interpretation and therefore rely heavily on radiology reports. When these reports do not identify adenomyosis, the opportunity for preoperative diagnosis is often missed. In our cohort of 124 patients, all were initially diagnosed with symptomatic fibroids by their referring providers and were subsequently referred to the Fibroid Clinic. Notably, none of these patients had been counseled about the possibility of adenomyosis before referral, highlighting a critical gap in preoperative evaluation and patient education. 

Our data indicated that AUB, dysmenorrhea, and dyspareunia are highly related to adenomyosis if the uterine size is less than 14 weeks' size, while UT is an independent factor for adenomyosis regardless of uterine size. Access to clinical information may have contributed to higher diagnostic suspicion during gynecologic review of imaging. Because imaging interpretation was not blinded and did not occur under standardized research conditions, this study was not designed to compare specialty-level diagnostic performance. In some cases, the MUSA features on TVUS were not complete or significant, which might be very vague for even experienced radiologists. However, with the clinical information, a MIGS might highly suspect an adenomyosis diagnosis even with minimal MUSA features on TVUS. However, we need to understand that the coexisting fibroids can significantly interference the diagnosis of adenomyosis in TVUS, which has been reported by several studies and reflected in our data as well [3,14]. Specifically, we observed that the sensitivity of MIGS-interpreted TVUS decreased from 83.3% in patients with pure adenomyosis to 41.0% when adenomyosis coexisted with fibroids. These results highlight that while TVUS is a valuable tool, but not completely reliable, even when performed or interpreted by experienced gynecologic specialists.

Our findings highlight key insights that may inform improved diagnostic and treatment strategies for this frequently overlooked condition. However, several limitations in this study need to be addressed. First, this study was a retrospective study conducted at a single community hospital. A similar research was done by Zanolli, et al. at a prestigious university hospital in 2022 [12]. Therefore, findings should be interpreted as hypothesis-generating and may not be generalizable beyond similar clinical settings. A multi-center study should be conducted to evaluate the accuracy of TVUS or MRI on adenomyosis by radiologists in real practice. Second, the sample size was small in this study. There were only 13 cases of pure adenomyosis and 50 cases of concomitant fibroids and adenomyosis. The selection bias cannot be completely excluded with a small sample size. Third, there were only five radiologists and one MIGS involved in this study. The five radiologists may reflect the real situation because there is no subspeciality of gynecology in radiology, but the one MIGS cannot represent the level of gynecology. Increased gynecologist involvement in imaging interpretation and physical examination may help bridge the diagnostic gap in practice.

## Conclusions

In conclusion, this study highlights the limitations of TVUS for diagnosing adenomyosis when imaging findings are interpreted without a comprehensive clinical context. Integration of patient history, pelvic examination, particularly uterine tenderness, and imaging findings may improve preoperative suspicion of adenomyosis. These findings support a multidisciplinary approach to diagnosis and underscore the need for enhanced gynecologic imaging education, interdisciplinary communication, and future multicenter studies to validate clinically integrated diagnostic frameworks.
